# Molecular Characterization of BsCu/Zn-Superoxide Dismutases and BsMn-Superoxide Dismutases from Chinese Black Sleeper (*Bostrychus sinensis*)

**DOI:** 10.3390/ani15050653

**Published:** 2025-02-24

**Authors:** Haolin Li, Suzhen Ran, Xiaoyu Wang, Bin Shen, Jianshe Zhang

**Affiliations:** 1 National Engineering Research Center of Marine Facilities Aquaculture, Zhejiang Ocean University, Zhoushan 316004, China; lihaolin324217@163.com (H.L.); wxy6661216@163.com (X.W.); 2 School of Foundation Studies, Zhejiang Pharmaceutical University, Ningbo 315211, China; ransz@zjpu.edu.cn

**Keywords:** Chinese black sleeper, Cd^2+^, innate immunity, gene expression, oxidation resistance

## Abstract

Superoxide dismutases (SODs) are critical antioxidant enzymes that protect organisms by catalyzing the dismutation of superoxide radicals into molecular oxygen (O_2_) and hydrogen peroxide (H_2_O_2_). In this study, we cloned the full-length cDNA of two SODs (BsCu/Zn-SOD and BsMn-SOD) from the Chinese black sleeper. The cDNA of BsCu/Zn-SOD consists of 465 nucleotides encoding 154 amino acids, while the BsMn-SOD comprises 678 nucleotides encoding 225 amino acids. The PCR results indicated that in normal tissues, the highest expression of BsCu/Zn-SOD (1.02-fold) was primarily observed in muscle, whereas the highest expression of BsMn-SOD (6.42-fold) was noted in skin. Following treatment with *Vibrio parahaemolyticus*, the highest expression of BsCu/Zn-SOD (3.67-fold) was detected in the spleen, while BsMn-SOD (3.36-fold) presented the highest expression in the blood. After poly(I:C) treatment, the highest expression of BsCu/Zn-SOD (2.92-fold) was found in the blood, with BsMn-SOD (2.37-fold) showing the highest expression in the spleen. Following Cd^2+^ treatment, the highest expression of BsCu/Zn-SOD (3.36-fold) was recorded in the liver at the 1/2 96 h LC50 concentration, and in the spleen at both the 1/4 96 h LC50 (2.50-fold) and 1/8 96 h LC50 concentrations. In contrast, BsMn-SOD presented the highest expression (2.60-fold) in the liver across all three concentrations. These findings suggest that both BsCu/Zn-SOD and BsMn-SOD play vital roles in the innate immune response to environmental challenges, laying the foundation for further exploration of antioxidant mechanisms.

## Simple Summary

In this study, the effects of cadmium (Cd^2+^) exposure and immune challenges on the antioxidant gene expression and immune response of the Chinese black sleeper were investigated. Cd^2+^ exposure caused variations in the expression of antioxidant genes, with peak expression occurring at different times in various tissues. Immune challenges with *Vibrio parahaemolyticus* and poly(I:C) induced dynamic changes in the expression of BsCu/Zn-SOD and BsMn-SOD, suggesting their roles in antioxidant defense and immune response. These findings provide insights into the physiological and immune responses of the Chinese black sleeper to environmental stress.

## 1. Introduction

Environmental pollutants derived from industrial, domestic, and agricultural activities pose significant threats to freshwater and marine ecosystems [1]. Among these pollutants, chemical contaminants, salinity changes, and heavy metals are known to induce the intracellular accumulation of reactive oxygen species (ROS) in aquatic organisms, leading to oxidative stress. ROS are highly reactive molecules that disrupt the oxidative-antioxidant balance, causing severe cellular damage [2]. Under normal physiological conditions, organisms maintain a dynamic balance of ROS through antioxidant systems, including key enzymes such as superoxide dismutase (SOD), glutathione peroxidase, and catalase [3,4]. However, excessive ROS generation can overwhelm these systems, triggering oxidative stress responses that are particularly detrimental to fish health.

SODs are crucial antioxidant enzymes that catalyze the dismutation of superoxide radicals into oxygen and hydrogen peroxide, providing the first line of defense against oxidative stress [5]. Based on the metal cofactors they contain, SODs are classified into four distinct types: Cu/ZnSOD, NiSOD, FeSOD, and MnSOD [6]. Cu/Zn-SOD is one of the most important free radical scavengers responding to oxidative stress, while MnSOD regulates toxin and pathogen responses in mitochondria [7]. Nowadays, there is a large amount of literature indicating that SODs are involved in protective responses of animals against oxidative stress induced by environmental challenges. Examples include species such as *Lates calcarifer* [8], *Sepiella maindroni* [1], and *Penaeus monodon* [9]. Collectively, these investigations underscore the critical importance of superoxide dismutase in the antioxidant enzyme system of fish.

The Chinese black sleeper (*Bostrychus sinensis*) is an economically significant marine aquaculture fish species in the southeastern coastal regions of China. Nowadays, this species is gradually becoming an important mariculture fish in the coastal areas of Zhejiang, Fujian, Guangdong, and Guangxi provinces in China [10]. However, with the growing scale of aquaculture, the Chinese black sleeper has been adversely affected by various environmental pollutants, leading to significant economic losses. Therefore, gaining a deeper understanding of the mechanisms underlying the immune response of *B. sinensis* to pathogen infections will aid in the development of strategies for disease management and contribute to the healthy growth of the mariculture industry for this fish species. In this study, we identified two SODs of *B. sinensis*, BsCu/Zn-SOD and BsMn-SOD, and analyzed their expression profiles in the Chinese black sleeper under cadmium stress and immune challenges.

## 2. Material and Method

### 2.1. Animals

Chinese black sleeper specimens were collected from a fish farm in Taizhou and acclimatized for two weeks before the experiments. A total of 120 healthy fish, weighing approximately 100 ± 50 g, were selected for the study and randomly distributed into experimental groups. Each experimental group consisted of 10 fish per tank, with three replicate tanks per treatment. The fish were maintained in a controlled environment with a water temperature of 24 ± 2 °C, a salinity of 12 ± 2‰, and a pH of 8 ± 0.5. They were fed a commercial diet containing 30% protein, and the lighting cycle was set to 12 h of light and 12 h of darkness. The water was filtered and changed regularly.

### 2.2. Median 96 h Lethal Concentration (96 h LC50) of B. sinensis Under Cd^2+^ Ion Stress

Acute cadmium toxicity tests were conducted by exposing animals to different concentrations of cadmium, following a pre-experiment test that progressively narrowed the range of concentrations between 0% and 100% mortality. CdCl_2_·2.5 H_2_O (HUSHI) was dissolved in deionized water at a concentration of 50 g/L to create a stock solution, which was then diluted to final concentrations of 50, 60, 70, 80, and 90 mg/L using deionized water. Each treatment contained 10 Chinese black sleepers, with three replicate tanks per treatment. A group with no cadmium exposure was set as the blank control. The static exposure method was employed under controlled conditions: temperature (17–19 °C), pH (6.9–7.5), and continuous aeration. During the experiment, feeding and water mixing were withheld to minimize external variables. Mortality rates were recorded at regular intervals, and probit regression analysis was used to calculate the 96 h LC50. The blank control group served as the baseline for comparison. The probit regression equation for the mortality rate of *B. sinensis* at 96 h was derived based on the model.

### 2.3. Cd^2+^ Stress Treatment

According to the 96 h LC_50_ values of Cd^2+^ for Chinese black sleeper, three concentrations (7.5, 15, and 30 mg/L), represented 1/8, ¼, and 1/2 of the 96 h LC50 value, were selected for the Cd^2+^ stress treatment. Tissues, including the liver, spleen, gill, skin, and muscle, were collected from the Cd^2+^-challenged fish at the following time points: 0, 3, 6, 12, 24, 48, 72, and 96 h post-exposure. For each time point, one fish was randomly selected from each of the three replicate tanks for replication.

### 2.4. Bacterial Infection and Poly(I:C) Stimulation

V. parahemolyticus (TZ-01) isolated from a diseased Chinese black sleeper was cultured in LB at 28 °C broth for 12 h and resuspended to 1 × 10^7^ CFU/mL in PBS [11]. The Chinese black sleepers were divided into two groups. For the experimental group, each fish was injected with 0.2 mL of bacterial resuspension. For the control group, 0.2 mL of sterile PBS was injected. Poly(I:C) stock (Sigma-Aldrich, St. Louis, MO, USA) was dissolved in phosphate-buffered saline (1xPBS) to the concentration of 1 mg/mL and filtered through a 0.22 μm filter. Intraperitoneal injections were administered at a dose of 250 mg/100 g. The control animals were injected with sterile 1xPBS of equal volume. Liver, spleen, head kidney, and peripheral blood were collected from both infected and control fish at the following time points: 0, 3, 6, 12, 24, and 48 h post-injection. Similarly, for each time point, one fish was randomly selected from each of the three replicate tanks for replication.

### 2.5. The cDNA Cloning of BsCu/Zn-SOD and BsMn-SOD

Total RNA was extracted using a standard RNA extraction kit and reverse-transcribed into cDNA using the Super Script™ III Reverse Transcriptase Kit (Invitrogen, San Diego, CA, USA). The specific amplification primers (Table 1) were designed using Primer Premier 5.0 software. PCR amplification was carried out using Premix Ex Taq™ (TaKaRa, San Jose, CA, USA) under the following thermal cycling conditions: initial denaturation at 95 °C for 5 min, 32 cycles at 95 °C for 30 s, 56 °C for 30 s, and 72 °C for 40 s.

### 2.6. Bioinformatics Analysis

DNAMAN 9 software was used for comparative analysis of the CDS regions of the DNA sequences. The prediction of amino acid functional structural domains was performed using the InterPro Scan 5 website (https://www.ebi.ac.uk/jdispatcher/ (accessed on 29 March 2024)). The amino acid homology of some of the antioxidant genes of the Chinese black sleeper was analyzed by Clustal W (Home|PBIL (ibcp.fr) (accessed on 29 March 2024)) and Blast (https://blast.ncbi.nlm.nih.gov/Blast.cgi/ (accessed on 29 March 2024)) for the amino acid homology of some antioxidant genes of Chinese black sleeper. MEGA 5.0 software and neighbor-joining were used to construct the phylogenetic tree. Isotopes and protein molecular weights were analyzed using the Expasy website (https://www.expasy.org/ (accessed on 30 March 2024)).

## 3. Result

### 3.1. Characterization of BsCu/Zn-SOD and BsMn-SOD

**Figure 1:**
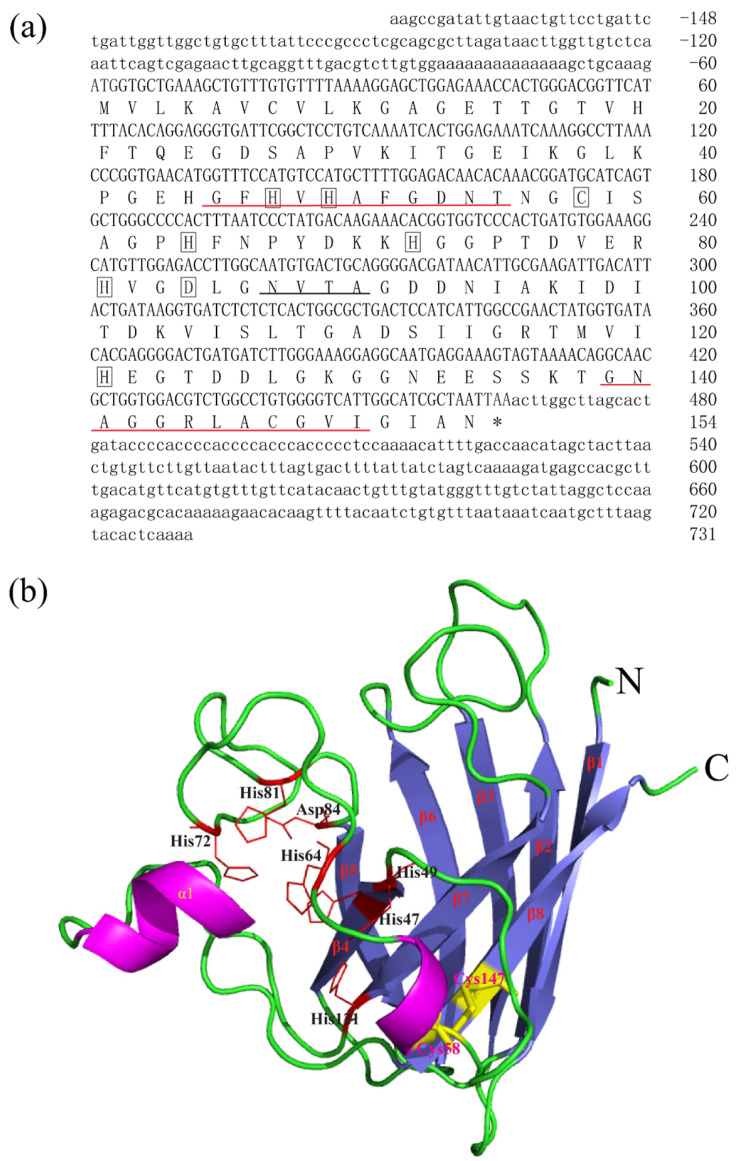
Nucleotide, the deduced aa sequences and 3D structures of BsCu/Zn-SOD. (**a**) Nucleotide and the deduced aa sequences of BsCu/Zn-SOD; nucleotides are numbered from the first base at 5′ end. Stop codons are marked with asterisks. Potential Cu^2+^-binding sites (His-47, -49, -64 h, and -121) and Zn^2+^-binding sites (His-64, -72, -81, and Asp-84) in BsCu/Zn-SOD were boxed. Putative family signature motifs are underlined in red. Po-tential N-glycosylation sites are underlined in black. (**b**) The predicted 3D structures of BsCu/Zn-SOD.

**Figure 2:**
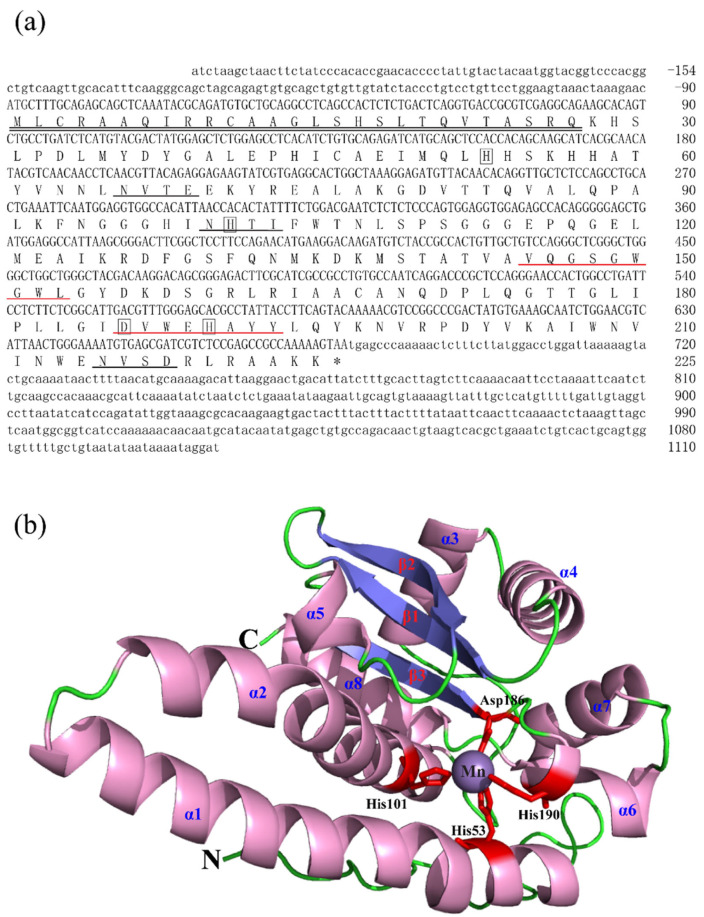
Nucleotide, the deduced aa sequences and 3D structures of BsMn-SOD. (**a**) Nucleotide and the deduced aa sequences of BsMn-SOD; Nucleotides are numbered from the first base at 5′ end. Stop codons are marked with asterisks; potential binding sites (His-53, -101, -190, Asp-186) in BsMn-SOD were boxed. Predicted MTS region is underlined with double underscores. Putative family signature motifs are underlined in red. Potential N-glycosylation sites are underlined in black. (**b**) The predicted 3D structures of BsMn-SOD.

The full-length cDNA sequences of BsCu/Zn-SOD and BsMn-SOD were sequenced and deposited in GenBank under Accession Nos. MT123896 and MT123897, respectively. The CDS sequence length of BsCu/Zn-SOD was 465 bp, encoding a protein that lacks a signal peptide, indicating its intracellular localization. Sequence analysis revealed a theoretical isoelectric point of 5.68, and a disulfide bond was identified between cysteine residues C^58^ and C^147^. Additionally, one putative N-glycosylation site was predicted (Figure 1). In contrast, the BsMn-SOD CDS consists of 678 base pairs, encoding a protein with a predicted molecular mass of 25.1 kDa and a theoretical isoelectric point of 5.68. Notably, a mitochondrial targeting sequence (MTS) comprising 27 amino acids was identified at the N-terminus, suggesting mitochondrial localization. Moreover, three potential N-glycosylation sites were predicted at positions ^66^NVTE^69^, ^100^NHTI^103^, and ^215^NVSD^218^ (Figure 2).

### 3.2. Effects of Cd^2+^ Exposure on 96 h LC50

In order to better study the toxic dose of cadmium from *B. sinensis*, we conducted a median lethal concentration (LC50) of cadmium on Chinese black sleeper by the probability unit method. The results of our median lethal concentration test showed that the Cd^2+^ exposure on 96 h LC50 in the Chinese black sleeper was 61.44 mg/L (54.42–70.09) in our experimental environment. The probit equation for the mortality rate of *B. sinensis* at 96 h was Y = −51.27 + 28.98X.

### 3.3. Homology and Phylogenetic Analysis

**Figure 3:**
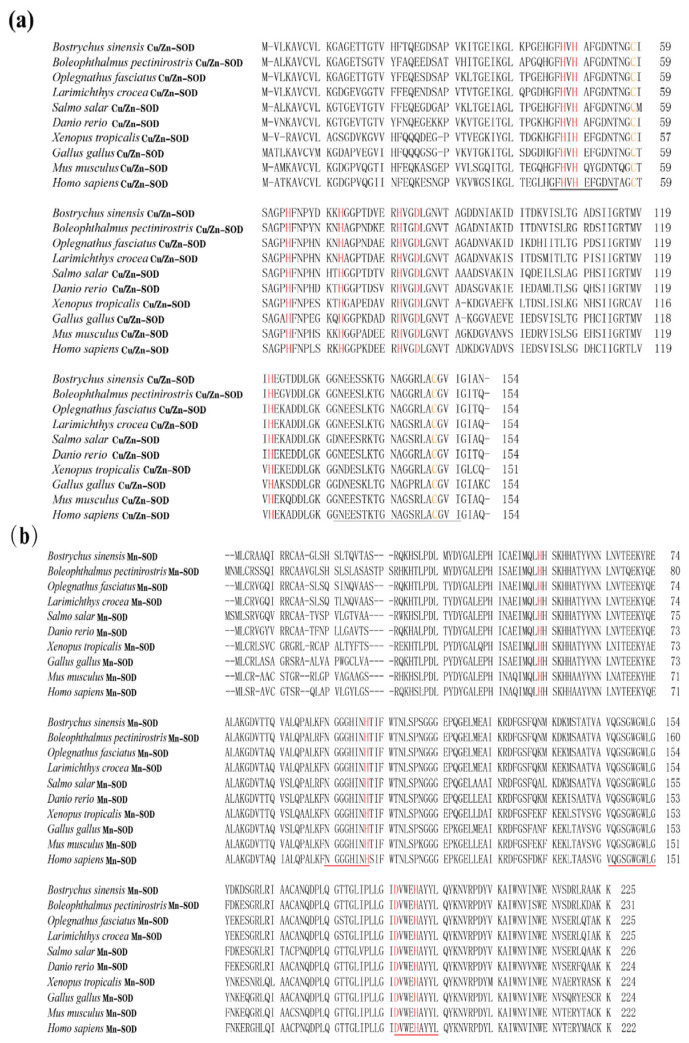
Sequence alignment of (a) BsCu/Zn-SOD and (**b**) BsMn-SOD genes from *B. sinensis.* The scale bar refers to the aa residues of (**a**) BsCu/Zn-SOD or (**b**) BsMn-SOD. Putative Cu^2+^ binding sites are shown in red. Conserved SOD-family signature motifs are underlined in (**a**) black and (**b**) red. C–C (disulfide bridges) are shown in yellow.

**Figure 4:**
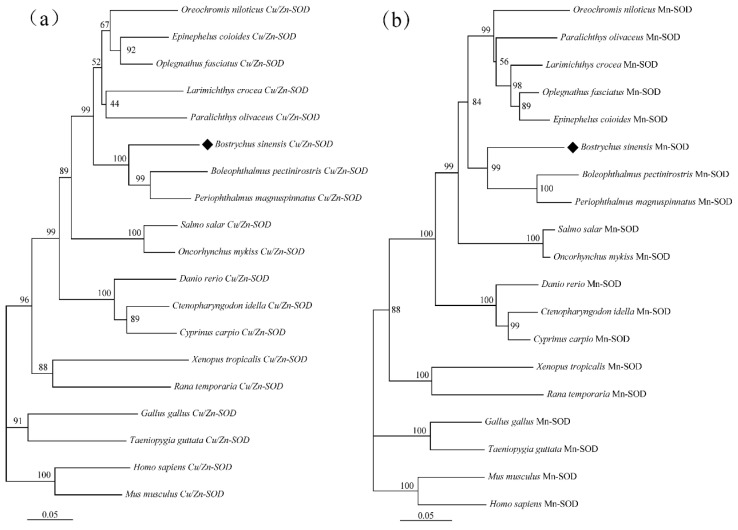
Hylogenetic tree of (**a**) BsCu/Zn-SOD and (**b**) BsMn-SOD in *B. sinensis***.** Phylogenetic tree based on SODs amino acid sequences using neighbor-joining (NJ) method, under the JTT model. Values on the nodes are NJ bootstrap values.

In the present study, the amino acid (aa) sequences of BsCu/Zn-SOD and BsMn-SOD were compared with those of SODs from different species (Figure 3). Alignment studies revealed the conserved regions in BsCu/Zn-SOD and BsMn-SOD, which include the signature motifs of the Mn and Cu/Zn families, invariant aa responsible for coordination of Mn and Cu/Zn, and two cysteines (C58 and C147). The sequence identity of BsCu/Zn-SOD and BsMn-SOD to their homologs averaged 75% and 80%, respectively. Phylogenetic tree analysis revealed that the Chinese black sleeper diverged from other phyla during the construction of the evolutionary tree for the BsCu/Zn-SOD and BsMn-SOD genes. Homology comparisons indicated that both BsCu/Zn-SOD and BsMn-SOD were derived from their respective common ancestors, similar to the homologous species of other scleractinian fishes (Figure 4).

In summary, BsCu/Zn-SOD and BsMn-SOD were evolutionarily conserved in protein sequences, functionally significant domains, and catalytic residues. This means that BsCu/Zn-SOD and BsMn-SOD may have significant roles in congenital immunity and oxidation resistance response, similar to teleosts.

### 3.4. Expression of BsCu/Zn-SOD and BsMn-SOD in Normal Tissues

**Figure 5:**
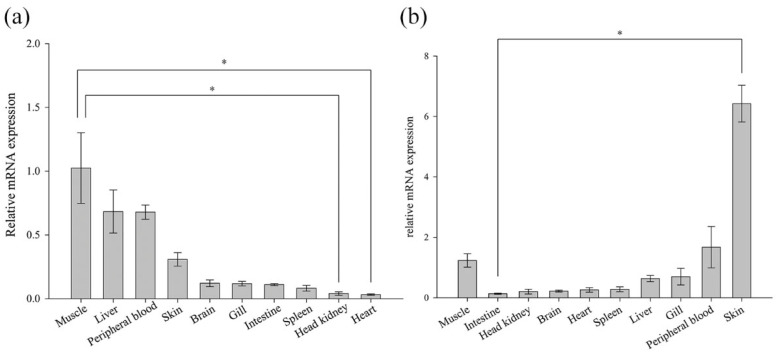
Expression of (**a**) BsCu/Zn-SOD and (**b**) BsMn-SOD genes in normal tissues. Gene expression of BsCu/Zn-SOD and BsMn-SOD in each examined organ was analyzed using the 2^−ΔΔCt^ method. Error bars indicate mean ± SD. * *p*-value < 0.05 is determined using one-way ANOVA followed by Tukey tests.

The expression analysis of ten normal tissues from the Chinese black sleeper was conducted using RT-PCR. The mRNA expression of both genes was detectable in the examined tissues (Figure 5). However, their relative levels of basal expression varied across different tissues. BsCu/Zn-SOD was predominant in muscle (1.02-fold), followed by liver (0.68-fold) and peripheral blood (0.68-fold). BsMn-SOD presented the highest expression level in the skin (6.42-fold), and the second-highest expression levels were found in the peripheral blood (1.678-fold) and muscle (1.23-fold).

### 3.5. Stimulation of BsCu/Zn-SOD and BsMn-SOD Expression by V. parahaemolyticus in the B. sinensis

**Figure 6:**
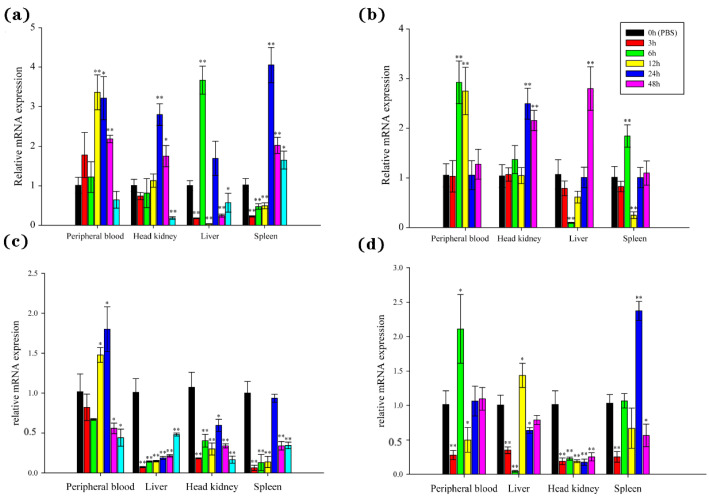
Expression analyses of the BsCu/Zn-SOD in various tissues following (**a**) *V. parahaemolyticus* and (**b**) Poly(I:C) stimulation, and the BsMn-SOD in various tissues following (**c**) *V. parahaemolyticus* and (**d**) Poly(I:C) stimulation. Three individuals were used for replication. Error bars show the standard deviation (SD). Statistical significance (** *p*-value < 0.01 and * *p*-value < 0.05) is determined by unpaired two-tailed Student’s *t*-Test.

The temporal expression witnessed explicit changes of BsCu/Zn-SOD and BsMn-SOD in examined tissues from 0 to 48 h after *V. parahaemolyticus* challenge. The expression levels of BsCu/Zn-SOD were quickly upregulated after bacterial infection, with peak expression observed at 6 h in the liver, 12 h in the peripheral blood, and 24 h in the head kidney and spleen. The peak levels reached 3.36-fold in the peripheral blood, 2.80-fold in the head kidney, 3.67-fold in the liver, and 4.05-fold in the spleen compared to the control group (Figure 6a). However, the expression levels of BsMn-SOD were quickly downregulated (except in the peripheral blood) after bacterial infection, with peak expression observed at 24 h in the peripheral blood and at 0 h (control) in the spleen, liver, and head kidney (Figure 6c).

The expression of BsCu/Zn-SOD and BsMn-SOD genes was significantly altered after stimulation by poly(I:C). The expression levels of the BsCu/Zn-SOD were significantly changed in the peripheral blood and head kidney, with the peaks appearing at 6 h in and 24 h. The peak levels reached 2.92-fold in the peripheral blood and 2.50-fold in the head kidney. The expression levels of BsCu/Zn-SOD showed the same trend in both the liver and spleen after poly(I:C) stimulation, with peak expression at 48 h in the liver and at 6 h in the spleen. The peak levels reached 2.80-fold in the liver and 1.84-fold in the spleen (Figure 6b). On the other hand, the expression levels of BsMn-SOD were quickly downregulated immediately after stimulation by poly(I:C), followed by a rebound in the peripheral blood, liver, and spleen, while inhibition persisted in the head kidney (Figure 6d). The peak expression occurred at 6 h in the peripheral blood, 12 h in the liver, 24 h in the spleen, and at 0 h (control) in the head kidney, with peak expression levels reaching 1.44-fold in the liver, 2.37-fold in the spleen, 2.11-fold in the peripheral blood, and 1.0-fold in the head kidney.

### 3.6. Expression of BsCu/Zn-SOD and BsMn-SOD Genes in Cd^2+^ Stress

**Figure 7:**
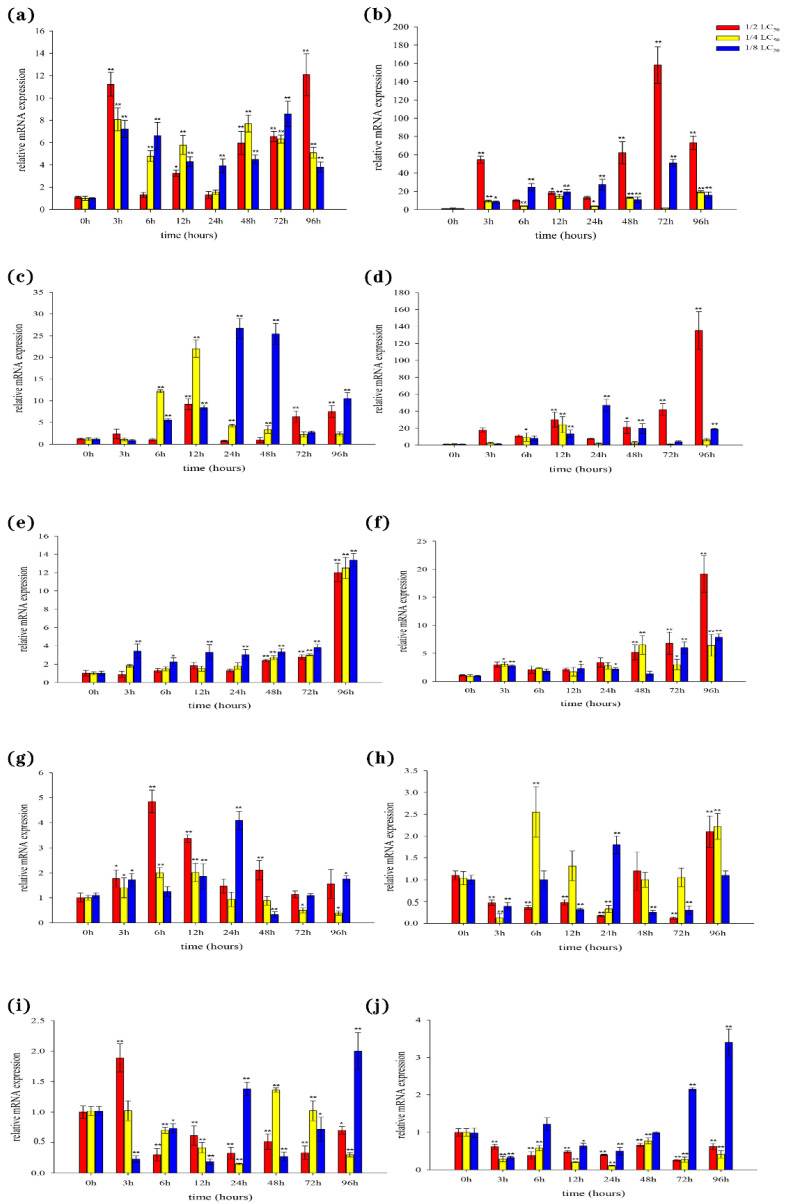
Expression analyses of the BsCu/Zn-SOD in (**a**) liver (**c**) spleen (**e**) gill (**g**) skin (**i**) muscle after Cd^2+^ challenge, and BsMn-SOD in (**b**) liver (**d**) spleen (**f**) gill (**h**) skin (**j**) muscle after Cd^2+^ challenge. Three individuals were used for replication. Error bars show the standard deviation (SD). Statistical significance (** *p*-value < 0.01 and * *p*-value < 0.05) is determined by unpaired two-tailed Student’s *t*-Test.

In the liver, our results showed that the expression levels of both BsCu/Zn-SOD and BsMn-SOD were quickly upregulated following Cd^2+^ stimulation (Figure 7a,b). However, the peak expression times of BsCu/Zn-SOD and BsMn-SOD varied at different concentrations of Cd^2+^ exposure. In the spleen, the expression levels of antioxidant response genes were significantly altered three hours after Cd^2+^ stimulation. The expressions of the BsCu/Zn-SOD in 1/2-LC50 and 1/4-LC50 peaks appeared at 12 h, while in 1/8-LC50 peaks appeared at 24 h (Figure 7c). The expression level of BsMn-SOD in 1/2-LC50 peaks appeared at 96 h, while in 1/4-LC50 and in 1/8-LC50 peaks appeared at 12 h and 24 h (Figure 7d). In the gills, expression of the BsCu/Zn-SOD and BsMn-SOD were significantly changed after stimulation by Cd^2+^ 72 h later (Figure 7e,f). The peak expression levels of BsCu/Zn-SOD and BsMn-SOD were observed at 96 h for all three Cd^2+^ concentrations. In the skin, the expressions of the BsCu/Zn-SOD in 1/2-LC50 and 1/4-LC50 peaks appeared at 6 h, while in 1/8-LC50, peaks appeared at 24 h (Figure 7g). The expression level of BsMn-SOD in 1/2-LC50 peaks appeared at 96 h, while peaks appeared in 1/4-LC50 and in 1/8-LC50 at 6 h and 24 h (Figure 7h). In the muscle, the expression profile changes of the BsCu/Zn-SOD and BsMn-SOD from Chinese black sleeper stimulation by Cd^2+^ were quite different. The peak from the expression of BsCu/Zn-SOD appeared at 3 h under 1/2-LC50, while in 1/4-LC50 and 1/8-LC50, peaks appeared at 48 h and 96 h (Figure 7i). However, the expressions of the BsMn-SOD in 1/2-LC50 and 1/4-LC50 are negative feedback. At the low concentration of 1/4-LC50, the peak expression of BsMn-SOD was observed at 96 h (Figure 7j).

## 4. Discussion

In this study, the sequencing and comparison of BsCu/Zn-SOD and BsMn-SOD genes, along with phylogenetic tree analysis, indicated that their characteristic sequences, metal-binding sites, and catalytic regions are highly conserved. Structural predictions indicated that BsCu/Zn-SOD mainly adopts a β-sheet, whereas BsMn-SOD primarily exhibits an α-helix configuration. These structural differences suggest that two genes may play different roles in antioxidation processes [12]. Phylogenetic analysis demonstrated that Cu/Zn-SOD evolved quickly and erratically, while Mn-SOD evolved much more regularly [13]. Some previous studies suggested that the evolutionary velocities of Cu/Zn-SOD and Mn-SOD were different, and Cu/Zn-SOD evolved erratically faster than Mn-SOD due to its efficient ability of protecting cells against oxygen toxicity [13,14]. The increased evolutionary rate of Cu/Zn-SOD may be linked to the rise in both terrestrial and marine pollution due to industrialization, which likely accelerated the evolution of Cu/Zn-SOD [15].

The tissue distribution analysis revealed that the mRNAs of BsCu/Zn-SOD and BsMn-SOD were ubiquitously expressed across all ten examined tissues, consistent with previous studies in other fish species. The SODs genes in other teleost fishes, such as *Pseudosciaena crocea* [9], *Hypophthalmichthys molitrix* [16], *Anguilla marmorata* [17], *Hemibarbus mylodon* [18], and *Liza haematocheila* [19], were all observed to be constitutively expressed in all examined organs. BsCu/Zn-SOD and BsMn-SOD were highly expressed in muscle and blood, similar to the SOD genes in other teleost fishes, such as *Anguilla marmorata* [17] and *Pseudosciaena crocea* [9]. SODs were highly expressed in the main immune organs: the liver, spleen, and gill. It is speculated that these highly expressed tissues have elevated concentrations of superoxide radicals (O^−^), and SODs may catalyze the conversion of superoxide radicals (O^−^) into hydrogen peroxide (H_2_O_2_) and oxygen (O_2_), protecting cells from ROS-induced cytotoxicity.

Bacterial infections are among the causes of fish diseases, and different species exhibit different expression patterns when faced with various external stimuli [20]. For example, following stimulation by *A. hydrophila* in *Anguilla marmorata*, Cu/Zn-SOD and Mn-SOD demonstrated a pattern of upregulation and then downregulation in various immune tissues, with the highest expression in the head kidney and lower levels in the liver [17]. In the present study, stimulation by *V. parahaemolyticus* in the Chinese black sleeper led to an initial downregulation of gene expression in some tissues. This was followed by upregulation before the expression returned to normal levels. The highest expression was recorded in the peripheral blood and spleen. This study revealed that the expression of certain antioxidant enzyme genes is tissue-specific and dynamic, showing spatiotemporal specificity. On the basis of these results, it can be inferred that the invasion of exogenous substances disrupts internal homeostasis, leading to an increased production of reactive oxygen species (ROS). In turn, it induces the expression of antioxidant genes in immune organs. In the early stages of infection, the expression of antioxidant genes in some immune organs may be lower than normal, possibly because of the damage caused by high levels of ROS to bodily functions. As time progresses and bodily functions recover, many antioxidant genes are expressed to eliminate ROS and restore homeostasis.

Cd^2+^ has emerged as one of the most prevalent heavy metal pollutants in coastal areas, resulting in irreversible damage to fish growth. The median lethal concentration (LC50) of Cd^2+^ varies among different species. The results of the analysis indicate that the acute toxicity of this heavy metal differs across various species and even similar species. For the same species, the LC50 for the same stressor can also differ under different experimental conditions within the same timeframe. The results of this study indicate that the Chinese black sleeper demonstrates higher Cd^2+^ resistance compared to other bony fish in terms of acute toxicity. This resistance in *B. sinensis* may be attributed to the robust regulatory function of its antioxidant genes [21]. The elevated levels of nonenzymatic cellular antioxidants relative to other fish species [22]. Additionally, the higher hardness of seawater compared to freshwater results in seawater fish absorbing fewer metal ions than freshwater fish under the same pollutant levels, leading to a higher LC50 for seawater fish [23]. Analysis of the results indicates that the overall trend of antioxidant gene expression in the immune organs of the Chinese black sleeper after Cd^2+^ exposure first increases and then decreases to normal levels. High expression levels in the liver and spleen regulate the production of superoxide dismutase, protecting the organism, which confirms the status of the liver and spleen as the primary immune organs in fish [24]. In muscle tissue, low concentrations of Cd^2+^ inhibit gene expression; however, during the middle to late stages of stress, a surge in expression is observed. These findings suggest that although low concentrations of Cd^2+^ initially suppress gene expression, a significant production of antioxidants is later induced to protect the fish, indicating that the damage to muscle tissue caused by low Cd^2+^ exposure is reversible. Heavy metal exposure in fish reduces mitochondrial enzyme activity in muscle tissue, but this activity can be partially restored through interactions among different enzymes [25]. These enzyme activities are associated with the antioxidant response to heavy metal toxicity in muscle tissue, suggesting that low to moderate concentrations of Cd^2+^ are relatively safe for Chinese black sleeper muscle.

The fundamental mechanism by which *B. sinensis* resists environmental pollutant stress is to ensure the scavenging of free radicals, thereby reducing the damage caused by free radicals to cellular biomacromolecules and maintaining normal cellular activities. Our results demonstrate that environmental pollutant exposure induces the expression of antioxidant system-related genes in the liver, spleen, gills, skin, and muscle of the Chinese black sleeper to varying degrees [26]. Environmental pollutant exposure induces the production of reactive oxygen species (ROS) within fish cells, and the upregulated expression of BsCu/Zn-SOD and BsMn-SOD leads to the production of enzymes and proteins that can significantly eliminate excessive ROS, thereby ensuring normal physiological functions. Furthermore, a comparison of BsCu/Zn-SOD and BsMn-SOD gene expression under Cd^2+^ stress revealed that BsMn-SOD expression in the five tissues of the Chinese black sleeper increased over time, with a more pronounced trend than BsCu/Zn-SOD expression [27]. However, in response to *V. parahaemolyticus* stress, the expression patterns of BsCu/Zn-SOD and BsMn-SOD are inversely related, suggesting that distinct immune mechanisms may govern the roles of BsCu/Zn-SOD and BsMn-SOD in terms of antimicrobial and antimetallic ions, which play different roles in the immune processes of fish.

## 5. Conclusions

This study successfully characterized the full-length cDNA sequences of BsCu/Zn-SOD and BsMn-SOD, revealing conserved structural features and evolutionary significance. Tissue expression analysis indicated that both genes are highly expressed in immune organs such as the liver and spleen. Upon Vibrio parahaemolyticus challenge, BsCu/Zn-SOD was significantly upregulated in immune tissues, suggesting its critical role in antibacterial defense, while BsMn-SOD showed dynamic regulation, indicating distinct functions in immune responses. Under Cd^2+^ stress, both genes exhibited tissue-specific expression, with the liver and spleen showing the highest levels, highlighting their antioxidant roles in mitigating oxidative damage. These findings suggest that BsCu/Zn-SOD and BsMn-SOD play vital roles in oxidative stress resistance and immune responses in *B. sinensis*, providing valuable insights for understanding stress adaptation and disease resistance in teleosts.

## Figures and Tables

**Table 1:** PCR primer sequences for BsCu/Zn-SOD and BsMn-SOD from *B. sinensis*.

Primer	Sequences
For complete ORF sequence	
BsCu/Zn-SOD-F	5′ CTGCAAAGATGGTGCTGAAAGC 3′
BsCu/Zn-SOD-R	5′ GCCAAGTTTAATTAGCGATGCCA 3′
BsMn-SOD-F	5′GTATCTACCCTGTCCTGTTCCTGG 3′
BsMn-SOD-R	5′ TATTTTGAATGCGTTTGTGGCTTG 3′
For qRT-PCR	
qBsCu/Zn-SOD-F	5′ CACAGGAGGGTGATTCG 3′
qBsCu/Zn-SOD-R	5′ GTGTTTCTTGTCATAGGGAT 3′
qBsMn-SOD-F	5′ GGCACTGGCTAAAGGAGATG 3′
qBsMn-SOD-R	5′ TCCACTGGGAGAGAGATTCG 3
β-Bsactin-F	5′ GACCCAGATTATGTTTGAGA 3′
β-Bsactin-R	5′ CGTGGTGGTGAATGAGTAG 3′

## Data Availability

The partial data analyzed in this study are available from the corresponding author upon reasonable request.
